# *T-REX*: software for the processing and analysis of T-RFLP data

**DOI:** 10.1186/1471-2105-10-171

**Published:** 2009-06-06

**Authors:** Steven W Culman, Robert Bukowski, Hugh G Gauch, Hinsby Cadillo-Quiroz, Daniel H Buckley

**Affiliations:** 1 515 Bradfield Hall, Department of Crop and Soil Sciences, Cornell University, Ithaca, NY, USA; 2 620 Rhodes Hall, Computational Biology Service Unit, Cornell University, Ithaca, NY, USA; 3 519 Bradfield Hall, Department of Crop and Soil Sciences, Cornell University, Ithaca, NY, USA; 4 Department of Microbiology, University of Illinois at Urbana-Champaign, Urbana IL, USA; 5 705 Bradfield Hall, Department of Crop and Soil Sciences, Cornell University, Ithaca, NY, USA; 6 3150 Plant and Environmental Science, Department of Land, Air and Water Resources, One Shields Avenue, University of California, Davis, CA 95616, USA

## Abstract

**Background:**

Despite increasing popularity and improvements in terminal restriction fragment length polymorphism (T-RFLP) and other microbial community fingerprinting techniques, there are still numerous obstacles that hamper the analysis of these datasets. Many steps are required to process raw data into a format ready for analysis and interpretation. These steps can be time-intensive, error-prone, and can introduce unwanted variability into the analysis. Accordingly, we developed *T-REX*, free, online software for the processing and analysis of T-RFLP data.

**Results:**

Analysis of T-RFLP data generated from a multiple-factorial study was performed with *T-REX*. With this software, we were able to i) label raw data with attributes related to the experimental design of the samples, ii) determine a baseline threshold for identification of true peaks over noise, iii) align terminal restriction fragments (T-RFs) in all samples (i.e., bin T-RFs), iv) construct a two-way data matrix from labeled data and process the matrix in a variety of ways, v) produce several measures of data matrix complexity, including the distribution of variance between main and interaction effects and sample heterogeneity, and vi) analyze a data matrix with the additive main effects and multiplicative interaction (AMMI) model.

**Conclusion:**

*T-REX *provides a free, platform-independent tool to the research community that allows for an integrated, rapid, and more robust analysis of T-RFLP data.

## Background

The high-throughput nature of terminal restriction fragment length polymorphism (T-RFLP) makes this technique amenable for generating comprehensive datasets in the study of microbial communities. Despite continued improvements, the analysis of these datasets still requires numerous steps and data manipulations in order to interpret the results. These steps often become obstacles to the analysis, as they are time-intensive and prone to user and analytical error. Currently, some of the greatest obstacles of T-RFLP data analysis are: i) distinguishing true peaks from noise, ii) aligning peaks across samples iii) creating a two-way data matrix of T-RFs by samples from tabulated raw data, iv) rapid manipulation of data matrices, and v) determining which multivariate analysis is most appropriate for a particular dataset. Collectively, these obstacles create research inefficiencies, reduce method standardizations and may limit the amount of information gained from the analysis overall. To address these obstacles, we have developed *T-REX *(**T-R**FLP analysis **EX**pedited), a free, web-based tool to aid in the analysis of T-RFLP data. In this paper, we introduce and outline the functions of *T-REX *and how it addresses each of the above obstacles.

Distinguishing true terminal restriction fragments (i.e., true peaks) from background fluctuations in fluorescence is often a major challenge in T-RFLP data analysis. The selection of a baseline threshold can dramatically affect the complexity of the community fingerprint and downstream analyses, resulting in signal loss or noise retention. A common procedure is to apply an arbitrary baseline threshold across all samples to delineate true peaks from noise [1-3]. However, this approach is less than optimal, as noise in a sample varies in proportion to the amount of DNA subject to analysis, causing variation in the proportions of signal to noise between profiles. Various approaches have been described to address this issue [2-5]. In particular, those that seek to objectively eliminate noise on a sample by sample basis, such as a variable percentage threshold [5] or recursively selecting true peaks based on standard deviations of peak areas [3] can be more effective at minimizing this bias.

While the base pair size of every T-RF is determined in relation to an internal size standard, sizing errors can occur due to random fluctuations, purine content, and fluorophores [6,7]. These analytical errors in determining fragment length can result in TRF-drift between samples, in which the same fragment is incorrectly assigned a different size in different samples. These errors are either ignored and treated as analytical error, corrected through painstaking manual alignment [1], or aligned using an automated approach [2,8]. However, to date, there have been no reports on the effects of these three approaches. Since most peak alignment software isn't integrated with downstream multivariate analyses, it is often difficult to determine the effects of this alignment on the overall interpretation of the data.

Multivariate statistical analyses are commonly required to interpret T-RFLP data and to examine the impact of environmental variables or treatments on microbial community composition. Raw T-RFLP data exported from Genemapper™, Peak Scanner™, or similar size-calling software is typically in a tabulated or listed format, where one column contains all the records for each variable (i.e., one column for all T-RF sizes, one column for all peak heights, etc.). However, these data often need to be formatted into a two-way data matrix to facilitate import into a statistical software package capable of analyzing multivariate data. The formatting of tabulated raw data into a data matrix is generally performed manually or with an application such as a pivot table in *MS Excel*, after samples have been labeled with information pertaining to the experimental design (sampling period, treatment, replicate number, etc.). These formatting approaches can be laborious and error-prone.

A thorough analysis of large T-RFLP datasets requires various data matrix manipulations, such as examining all three types of data (presence/absence, peak height, peak area), relativization of peak height or peak area, averaging replicated samples, examining specific experimental factors, deleting spurious T-RFs, etc. Most spreadsheet software applications aren't amendable to these more sophisticated manipulations, making an exhaustive analysis of these data difficult. In our experience, the rational exploration of T-RFLP data, which properly accounts for experimental design, replication, and differences in signal to noise ratios can reveal patterns in ordinations that are obscured in less complete approaches to data analysis [9,10].

Finally, there is a lack of consensus in the literature today about which statistical analyses are more appropriate to analyze T-RFLP data. In a comparative study of multivariate methods, Culman et al. [10] reported that the sample heterogeneity and percent interaction effects of a T-RFLP dataset can be used as criteria to select the appropriate statistical approach for data analysis. Although sample heterogeneity can easily be calculated, the calculation of interaction effects is more algorithmically involved. Culman et al. [10] also demonstrated the utility of the Additive Main Effects and Multiplicative Interaction (AMMI) model as a robust and advantageous method for T-RFLP analysis. This model is found in only a few multivariate software packages offered today.

### Currently Available Software for T-RFLP Analysis

Currently, there are few options to choose from when analyzing T-RFLP microbial community data. Most software that has been developed is aimed at referencing T-RFLP profiles with a sequence database (e.g. TAP-TRFLP [11,12], MiCA [13], PAT [14], TRAMPR [15]. There are, however, a few available packages that do aid with exploratory multivariate data analysis. T-Align [8] implements an algorithm to align peaks, hence reducing the potential for subjective bias during peak alignment. Another package, T-RFLP Stats [3] allows users to align peaks (as does T-Align), group samples based on various classification procedures and then reference these profiles to a clone library. However, a drawback is that this software is written in three separate languages (R, Perl and SAS) requiring three separate platforms. These platforms are all primarily command line driven and can be cumbersome to inexperienced users. SAS also requires a purchased license for use. In addition, T-RFLP Stats offers no labeling procedure to designate and format raw data, nor does it perform any ordination analyses, argued by some to be superior to classification procedures for the exploratory analysis of microbial community data [16]. A few commercial software packages have become available in recent years that offer a range of features regarding electropherogram manipulation, with some limited multivariate procedures, most notably GelQuest (SequentiX, Germany), Genemarker (SoftGenetics, USA), and Torast (Dresden, Germany). However, the high costs of these programs make them inaccessible to some research labs. In addition, features and functions vary widely between these programs, as most were not primarily designed to facilitate T-RFLP analysis.

We developed *T-REX *to address current obstacles encountered in T-RFLP data analysis. We sought to build a program that integrated pertinent functions to streamline T-RFLP analysis. *T-REX *allows users to i) label raw data with attributes related the experimental design of the samples, ii) determine a baseline threshold for identification of true peaks over noise, iii) align T-RFs in all samples (bin T-RFs), iv) construct a two-way data matrix from labeled data and process the matrix in a variety of ways, v) produce several measures of data matrix complexity, including the distribution of variance between main and interaction effects and sample heterogeneity, and vi) analyze a data matrix with the AMMI model. *T-REX *offers users a consolidated, flexible and rapid analysis of T-RFLP data.

## Implementation

**Figure 1:**
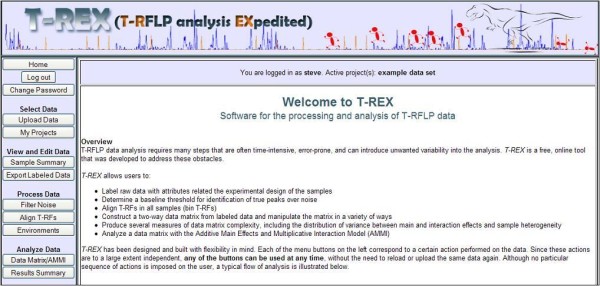
**Screenshot of *T-REX *Home page**.

**Figure 2:**
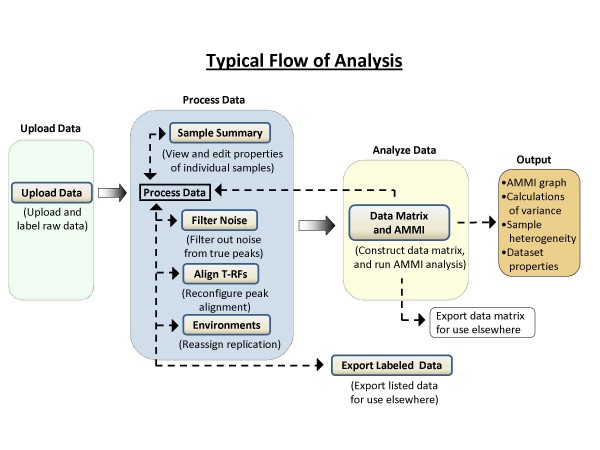
**Typical flow of project analysis in *T-REX***.

*T-REX *is a web-based application found at the address: . The program is free and requires only a web browser and an internet access to use. The home page outlines the program's features and introduces the user to a template of menu buttons (Figure 1). These menu buttons correspond to a certain action performed on the data. Since these actions are to a large extent independent, any of the buttons can be used at any time, without the need to reload or upload the same data again. Although no particular sequence of actions is imposed on the user, a typical flow of analysis is illustrated in Figure 2. Users can work as a guest or may become a registered user, for increased functionality.

### Uploading Data and Labeling Procedure (*Upload Data *and *My Projects*)

The first step in using *T-REX *is to create a project. A new project is created by uploading and labeling raw data. This process happens simultaneously and requires two files: i) the raw data file and ii) the label file. The raw data file is the tabulated file that is exported in GeneMapper^®^, PeakScanner™, or similar size-calling software that contains the peak information for a set of samples. The label file contains a set of labels/attributes that describe each sample and often correspond to factors in the experimental design. Both files should be simple text files in tab-delimited format (see the *T-REX *documentation for specific guidelines on file formats). Once a project is created, it can be renamed, merged, or deleted in the **My Projects **page. Users can also come back to pre-existing projects and load them in this page for further manipulation.

*T-REX *has several functions to appropriately handle replicated, missing or multiplexed [17] T-RFLP data. Users can define what samples are replicates when uploading data (or manually in the **Sample Summary **page) and *T-REX *will provide information based on these defined replicates. Missing data occurs when there is a discrepancy between samples in the raw data and label files, or when poor quality samples are flagged due to data processing procedures. *T-REX *accounts for missing data, allowing users to omit samples of poor quality without sacrificing information replicated data provide. In addition to replicated and missing data, *T-REX *is amenable to multiplexing T-RFLP methodologies. If a sample contains multiple fluors, peaks of the same fluor are processed as a unit of peaks, keeping them distinct from peaks of other fluors. The program documentation outlines specific guidelines for dealing with replicated, missing, or multiplexed data.

### Viewing and Editing Individual Samples (*Sample Summary*)

**Figure 3:**
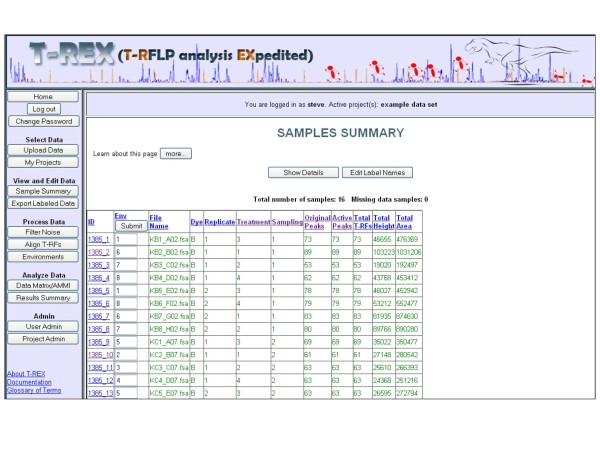
**Screenshot of T-REX Samples Summary page**.

The **Sample Summary **page is synonymous with the home page of a particular project (Figure 3). All samples are consolidated to show the total number of peaks, total peak height and peak area, as well as the properties relating to the experimental factors assigned in the labeling procedure. The **Sample Summary **page also shows users how data processing procedures (such as noise filtering or T-RF alignment) have removed peaks originally found in the raw data file.

**Figure 4:**
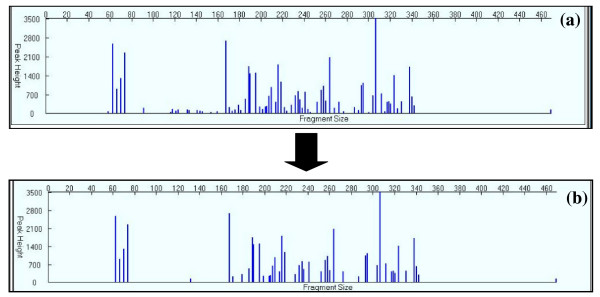
**Screenshot of Sample Details page in *T-REX***. Screenshot of Sample Details page in *T-REX*, before noise filtering function was applied to the sample (a) and after the noise filtering function was applied (b).

Individual samples can be viewed, edited, and even removed from the analysis in the **Sample Details **page, accessible by selecting the sample **ID **in the **Sample Summary **page. Once viewing an individual sample, the user will see individual peak properties and will be able to manipulate labels, remove individual peaks of that sample, or mark the entire sample as missing data within the project. The **Sample Details **page also allows users see the effects of data manipulation on the reconstructed electropherograms of samples (Figure 4).

### Filtering out Noise from True Peaks (*Filter Noise*)

*T-REX *uses the approach outlined by Abdo et al. [3] to find true peaks and reduce background noise. True peaks are identified as those whose height (or area) exceeds the standard deviation (assuming zero mean) computed over all peaks and multiplied by the factor specified in the box provided. The procedure is then reiterated with the peaks which were not identified as true ones. The iterations continue until no new true peaks are found. The noise filtering can be applied to all samples or just selected samples in the active project. Users should select an appropriate standard deviation multiplier based on the original electropherograms and results of the filtering procedure. The program allows for rapid manipulation of the multiplier and subsequent reviewing of results in the **Samples Summary **page if a user wants to determine an appropriate multiplier empirically (Figure 4). At any time the filtering procedure can be cleared and the data reverted to their original state with the 'Clear filtering' button.

### Automated Alignment of Peaks (*Align T-RFs*)

Peak alignment in *T-REX *is performed on the set of currently active peaks and occurs automatically whenever this set changes as a result of data manipulation by the user. *T-REX *offers users two functions to align peaks in the **Align T-RFs **page. With the default option ('Round to the nearest integer'), peaks are simply rounded to the nearest nucleotide (integer) size. Alternatively, an automated alignment of peaks across all samples is also possible. This function models the approach taken by the software program *T-Align *[8]. Briefly the smallest peak across all samples is identified and tagged. Peaks within the range specified by the clustering threshold are then identified and grouped into a T-RF. The next smallest peak across all samples not falling into the first T-RF is identified and tagged. Peaks within the specified clustering threshold are identified and grouped with the second T-RF. This process continues until all peaks are grouped into a T-RF.

### Grouping Samples into Environments (*Environments*)

The **Environments **page allows users to rapidly classify samples into environments based on the given labels. This approach is especially useful when replication in an experiment occurred at multiple scales (e.g., analytical, field) and a user wants to compare results based these different ways of defining replication. Users can assign and/or reassign replicated samples into environments by using the provided checkboxes to define the set of labels that determine an environment. Samples will be considered replicates (i.e., belonging to the same environment) if they have identical sets of checked label values. The **Environments **page can be used as an alternative to specifying replicates at data upload, or to change the environment assignments made at the upload stage.

### Export Labeled Data to Use Elsewhere (*Export Labeled Data*)

The **Export Labeled Data **page was designed for users who want to take advantage of *T-REX*'s rapid labeling procedure and data manipulation functions, but analyze their data with another software program. After data are uploaded and labeled, users can export the labeled data directly, or can manipulate the data before exporting. The **Sample Summary **page indicates the current status of a project and will reflect the exact details of the data that will be exported.

### Data Matrix Construction and AMMI analysis (*Data Matrix/AMMI*)

The ***Data Matrix/AMMI ***page allows users to first construct a two-way data matrix and then run the AMMI model on this data matrix. Data matrix construction involves six steps. The first two steps require that all peaks be assigned to a particular T-RF and that each sample be associated with an environment. Typically, both these conditions are automatically satisfied and require no special action. The third step allows users to specify which type of data to use for data matrix construction (presence/absence, peak height or peak area), and if these data are to be averaged across replicates and/or relativized within samples. The fourth step allows users to select which experimental factors should be included in the data matrix. Users have the option of selecting all, or only a subset of specific fluors and/or factors to be included in the data matrix and subsequent analysis. The fifth step allows users to omit rare T-RFs or samples with poor peak representation.

T-RFs can be omitted based on number or percentage of occurrences across samples. Total number of T-RFs or the cumulative peak height or area can be used to eliminate certain samples. This T-RF and sample filtering step represents a final quality control on the resulting data matrix. Selecting 'Create Data Matrix' in the sixth step will take the user to another page where a data matrix is ready for download, and various data matrix properties are displayed, including total numbers of samples and T-RFs present, the maximum, minimum, and average number (average richness) of T-RFs across samples, and sample heterogeneity.

At this point the user is able to export the data matrix for analysis with another software package, or continue with the AMMI analysis by clicking 'View AMMI Analysis'. Choosing the latter will take the user to another page where a scatterplot displays the AMMI ordination scores and four output tables summarize results. The first table reports the full ANOVA table; the second table reports the estimations of interaction sum of squares (SS) for pattern and noise, if the data are replicated. The third table reports the percentages of variation from each main effects and interaction source. The fourth table shows the percentage of interaction signal variation that is captured by the first interaction principal components axes (IPCA). Several output files are also generated and available to download from this page. The specifics of these files are outlined in Table 1 and the details of this page are described more fully in the *T-REX *documentation.

**Table 1:** Files types generated by *T-REX *that are available to download.

**File Type**	**File Extension**	**Recommended Application**	**Function**
Essential Files:			
AMMI summary	.mm_sum	word processor	ANOVA calculations (Tables one – four)
AMMI Graphing Data	.mm_grph	spreadsheet	Environment and T-RF scores for graphing
Data Matrix	.matrx	spreadsheet	Data matrix for additional analyses with other software
Transposed Data Matrix	.tmatrx	spreadsheet	Data matrix for additional analyses with other software
Other Files:			
MATMODEL output file	.mm_out	word processor	Full MATMODEL output
MATMODEL input file	.mm_in	word processor	MATMODEL input file
Environments Assigned to Samples	.env	spreadsheet	Defines which samples are replicates
Labeled Data (list format)	.label	spreadsheet	Labeled raw data
All Files:			
Zipped folder containing all files	.zip	compatible .zip extractor	Archive of all output files

### Summary of Results and Output (*Results Summary*)

The **Results Summary **page reports the results of relevant basic data matrix properties and summarizes the results of the AMMI analysis in one place. The 'T-RF Abundance table' reports the number of samples (samples present) and percentage of samples (% of samples present) in which each T-RF occurs. All generated output files are also available for download at this page.

### Example Dataset

We used *T-REX *to analyze 16S T-RFLP data generated from soils under two different management histories–harvested tallgrass prairie and adjacent agricultural fields–from five different sites across north central Kansas (Culman et al., unpublished). Soil was sampled at 3 different depth intervals (0 – 10 cm, 10 – 20 cm, and 20 – 40 cm) in June 2007. T-RFLP procedures were conducted as previously described [9]. The data were subjected to several quality control procedures–T-RF Alignment (clustering threshold = 0.5), Noise Filtering (peak area, standard deviation multiplier = 1) and elimination of samples with less than 20 T-RFs. This initial processing deemed that all 30 samples were of good quality and suitable to include in the final ordination analyses. Processed data were subject to the AMMI analysis with *T-REX *in two separate ways–first, with data defined as un-replicated (3 depths × 2 management histories × 5 different sites) and second with each site defined as a replicate (3 depths × 2 management histories × 5 replicates). Analyzing data as un-replicated was performed to gain insight into variability between sites; a second analysis with sites defined as replicates allowed for a more focused analysis on the experimental factors of primary interest–management history and depth. With replicated data, the AMMI analysis provides a calculation of interaction pattern and noise, providing a more resolute picture of the strength of the interaction term.

Defining replication was easily performed in the **Environments **page. Sample heterogeneity calculations provided by *T-REX *were high relative to T-RFLP datasets previously encountered [10]. As a result, we also used nonmetric multidimensional scaling (NMS) to analyze the data. The *T-REX*-constructed data matrices were then exported and subjected to NMS in R [18] via the *metaMDS *function in the *vegan *package. NMS parameters were manipulated in a variety of ways, but the final analyses were performed with *metaMDS *default parameters with the following exceptions: autotransform = false, 100 runs. NMS ordination results were graphed in R. After observing the AMMI ordination results in the scatterplot provided by *T-REX*, graphing scores were exported and graphed in R for publication purposes.

In addition to ordination results, the AMMI analysis provides a breakdown of the contributions of variation from the three sources in the data matrix, i) T-RFs, ii) environments, and iii) T-RFs × environments interactions. The variation from T-RFs reflects variability in the means of different T-RFs, while the variation from environments reflects the number of peaks or overall signal strength in T-RFLP profiles. The variation from T-RFs × environments interactions reflect how T-RFs differentially respond with the environments. For our research objectives, the interaction variation was the source of primary interest, as we were concerned with the response of microbial community profiles (T-RFS) to different depths and management histories (environments). Culman et al. [10] found that variation due to interaction effects reflect how similar or dissimilar the microbial communities are, and could be used as a tool to objectively assess differences across multiple datasets.

## Results and discussion

**Figure 5:**
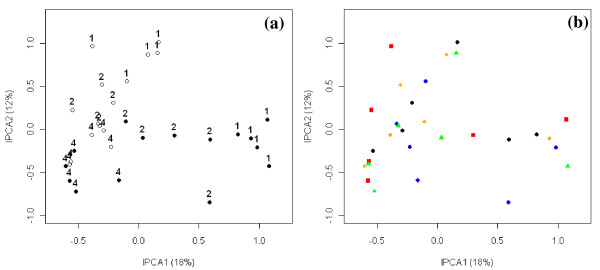
**AMMI analysis demonstrating differences in TRFLP patterns due to depth and management history (a) or site (b)**. TRFLP profiles with each site treated as an individual sample without replication. The two panels show the same data, but labeled with different symbols, illustrating differences due to (a) depth and management history and (b) site. In panel (a), closed circles represent agricultural soils; open circles represent prairie soils. 1 = 0 – 10 cm; 2 = 10 – 20 cm; 4 = 20 – 40 cm. In panel (b), different colors and symbols represent the five sites.

**Figure 6:**
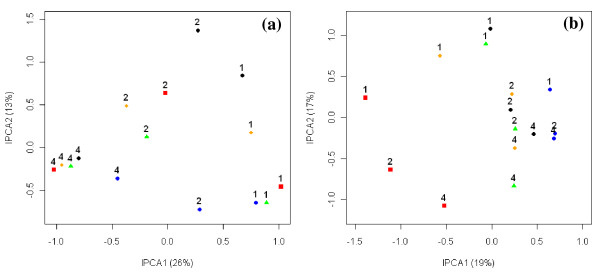
**AMMI analysis of bacterial T-RFLP datasets from agricultural (a) and prairie (b) soils**. TRFLP profiles were examined within management history, thereby eliminating variance due to differences in management. Each site was treated as an individual sample without replication to examine the amount of variance due to site within each management history. Different colors and symbols represent the five sites from agricultural (a) and prairie (b) soils. 1 = 0 – 10 cm; 2 = 10 – 20 cm; 4 = 20 – 40 cm.

Analysis of all the samples in the dataset revealed that depth was the primary driver of bacterial community structure (Figure 5a), as this factor was captured by the first interaction principal component (IPCA1). Differences in management history (prairie vs. agriculture) were secondary drivers of community structure, separating out on IPCA2. Some differences between sites could be observed, but no consistent trends were apparent in this ordination (Figure 5b). Although sample heterogeneity was high for this dataset (2.5), NMS analyses proved to be no more discriminatory than the AMMI analysis in revealing trends (data not shown). The data were further analyzed by examining the relationship of depth with each management history individually. Samples from prairie soils were easily removed from the dataset by selecting only the agriculture factor during the data matrix construction in *T-REX*. Analysis of the agricultural samples alone revealed similar trends with depth (Figure 6a) and resulted in a sample heterogeneity of 1.86. Differences in sites were also observed, as values of the IPCA 2 loosely reflected differences in sites. The analysis of bacterial communities from prairie soils revealed that differences in site were the primary driver of community structure in these soils (IPCA1), while differences in depth were a secondary driver (IPCA2; Figure 6b). Sample heterogeneity in the prairie dataset yielded was 1.55.

Analysis of the sources of variation of the un-replicated dataset revealed that bacterial communities from agricultural soil had the largest variation from interaction effects (58.4%, Table 2). Redefining the dataset so that each site was a replicate resulted in a decomposition of the interaction effects into pattern (signal) and noise. This approach again revealed that the agricultural soil had the greatest variation of interaction effects (32.7%), which were composed of 21.5% pattern and 11.2% noise (Table 2). In contrast, the bacterial communities from the prairie sites had a relatively low interaction pattern of (7.9%) and high interaction noise of 11.6%, indicating the majority of the interaction was idiosyncratic noise. The much larger contribution of interaction signal in agricultural sites compared to prairie sites, indicates that bacterial communities in these soils shared greater differences among samples than did the bacterial communities in prairie soils.

**Table 2:** *T-REX *output of the percent variation from each source in the three datasets.

	**Source**	**Agricultural + Prairie Soil**	**Agricultural Soil**	**Prairie Soil**
**Not replicated**				
	Main Effects			
	T-RFs	40.7	36.6	41.3
	Environment	4.1	5.0	7.2
	Interaction			
	Total	55.2	58.4	51.5

**Replicated**				
	Main Effects			
	T-RFs	63.6	61.3	70.3
	Environment	5.0	6.0	10.2
	Interaction			
	Pattern	19.9	21.5	7.9
	Noise	11.6	11.2	11.6

The T-RFLP dataset in this study contained three factors (depth, management history, and site), all of which were detectable drivers of bacterial community structure. However, the strength of site differences varied depending on management practice. Hence, exploratory data analyses and data matrix manipulation were required to elucidate which factors exerted the greatest influence on bacterial community structure within a specified treatment. *T-REX *aided in an integrated and rapid manipulation of these data matrices, enabling a thorough analysis of this dataset.

In addition to rapid data matrix manipulation, *T-REX *also produced a more robust dataset, as prior to data matrix construction, the data were subjected to several quality control procedures–T-RF Alignment, Noise Filtering, and elimination of samples with less than 20 T-RFs. This initial processing ensured that all samples were of acceptable quality. The calculations of sample heterogeneity and interaction effects generated by *T-REX *were also used as prescriptive indicators that the data were complex and that non-parametric analyses, such as NMS, may yield more discriminatory ordination results. However, the overall trends revealed by NMS did not differ from the ordination results of the AMMI analyses (not shown).

## Conclusion

*T-REX *facilitates an integrated and streamlined analysis of microbial community data with a suite of flexible functions that allows researchers to choose the most appropriate data manipulations based on research objectives. *T-REX *also enables researchers to implement the AMMI analysis, a method which holds many advantages for microbial community data analysis. In addition, this software provides a tool to the research community to rapidly and robustly test the effects of various data processing methods on the overall results of datasets. Many of these processing methods are known sources of analytical variability, but there is no consensus in the literature of how to most appropriately minimize this variability. We intend to focus the continued development of *T-REX *on a more sophisticated T-RF alignment algorithm, as well as integrating NMS and permutational multivariate analysis of variance. *T-REX *will allow microbial community analyses to continue to develop as an important tool in understanding microbial community dynamics and their effects on ecosystem processes.

## Availability and requirements

- **Project name**: *T-REX*

- **Project home page**: 

- **Operating system(s)**: Platform independent for users

- **Programming language**: Microsoft ASP.NET and MS SQL Server platforms

- **License**: GNU GPL

- **Any restrictions to use by non-academics**: none

## Authors' contributions

SC contributed to all sections of the software's development and drafted the initial manuscript. RB wrote all of the source code and contributed to the software's overall development. HG contributed to sections on the AMMI analysis, the ANOVA calculations, and the integration of MATMODEL. HCQ contributed to the development of data processing procedures and helped with testing of the software's functions. DB provided counseling on issues related to data processing and analysis, and guided the project's development. All authors contributed to the writing of the manuscript.
